# 

**Published:** 1817-08

**Authors:** 


### BOOKS IN THE PRESS.

Dr. BLAKE has nearly ready for the press, a volume in 12mo. of Practical Aphorisms, illustrating simple and complex Cases of Accouchement, Uterine Hæmorrhage, and Puerperal Peritonitis.

Dr. BANCROFT has in the press, and nearly ready for publication, A Sequel to his Essay on Yellow Fever.

In the press, and speedily will be published, The History of Vaccination; by JAMES MOORE, Surgeon.

In the press, and nearly ready for publication, the second edition, corrected and enlarged, of, Practical Observations on the Cure of the Gonorrhœa Virulenta in Men; by THOMAS WHATELY, Surgeon.

---

*We cannot account for the backwardness of the medical booksellers in furnishing us with the* Catalogue of Books *published during the month. We promise our readers, that, in future, they shall not be disappointed in this respect.—We shall be thankful for notice from authors.*

---

### *NOTICES TO CORRESPONDENTS.*

*We are much obliged to* MENTOR *for his kind intentions, and still more flattered by his preferring our Critiques to Original Correspondence. We think him, however, a little fastidious in objecting to a controversy which scarcely occupies three pages out of five hundred and thirty-six pages of our last volume. We are ignorant to whom he refers by his* coarser *inuendos.*

*Several favors are received, to which due attention shall be paid.*

